# A decade of GigaScience: A perspective on conservation genetics

**DOI:** 10.1093/gigascience/giac055

**Published:** 2022-06-14

**Authors:** Stephen J O'Brien

**Affiliations:** Halmos College of Arts and Sciences, Nova Southeastern University, Ft. Lauderdale, Florida 33004, USA

## Abstract

Wide interest in species conservation is young. To many it began early in 1903 when Teddy Roosevelt and John Muir set up a camp under the Grizzly Giant in the Mariposa Grove of California's Yosemite Valley. Over three days they decided to broaden the US National Park footprint across the USA. Conservationists were inspired in the coming decades by the writings of wildlife conservation pioneers—Osa Johnson (*I Married Adventure*), Karen Blixen (*Out of Africa*) and Rachel Carson (*The Silent Spring*). Countless crusaders developed a passion for preserving dwindling species in those early days, yet none of these conservation advocates mentioned the word genetics, let alone genomics. The genome sequencing projects that have followed on from these have brought in an enormous amount of data, including whole genome sequences for thousands of non-human species, both individual and population wide. This huge resource has revolutionized conservation genetics, bringing in ways to assess the health of at-risk populations, devise genetic-driven breeding strategies, and other means to attempt to preserve the over 1 million species (and growing) under threat today.

## Background

The genomics era began in 1986: It was around the time the word “genomics” was coined in a Bethesda saloon. After a tiring day of conferences at the annual Human Gene Mapping Workshop, Jim Womack, Tom Roderick, Tom Shows and I retired to the nearby MacDonald's Raw Bar. Academic Press had decided to launch a new scientific journal to highlight the powers of human genetics. The publishers sought our advice on the advisability, a potential editor, and the name of the new journal. The editor was easy: we suggested Victor McKuisick, a pathfinder in human genetics development. The Journal name was tricky. “Genome” seemed a great choice, but Tom Shows pointed out that the Canadian Journal of Genetics and Cytogenetics had just changed its name to “Genome.” Puzzled and frustrated, we spent a few pitchers of Pabst Blue Ribbon trolling for a name that fit. Once a pregnant pause came on, Tom Roderick, an affable and adroit mouse geneticist from Jackson Laboratory, took a deep breath and quietly mused “… *how about Genomics*?.” Silence befell us as all as we considered this odd-sounding brand new utterance. Within some moments we agreed: we all loved it. McKuisick would embrace it as did his co-editor, the prescient Frank Ruddle. The new term **Genomics**, first as a journal title and soon as a whole new science discipline, was born.

In those days, there was considerable optimism that an unabridged human genome DNA sequence could change everything in genetic medicine, from neuroscience to pharmacogenomics to personalized medicine. Full genomes would also invigorate forensics power, ancestry assessment, and comparative genomics of species. When the first draft of a human genome appeared in 2001, we quickly learned about genome size and structure, repeat complexity, gene distribution, pseudogenes and embedded DNA variants. Yet there were countless new questions about gene action, interaction, molecular evolution patterns, regulation, and development. New genomic disciplines were spawned that were only hinted at before the 21^st^ century. The quantum leap in subjects, applications, and innovations in genomic papers—along with the flood of data—led to the founding of *GigaScience* a decade ago.

*One of the new opportunities involved the challenges of species conservation*.

Genetics was first appreciated by Charles Darwin's recognition that close inbreeding in farm animals caused “inbreeding depression” due to increased expression of deleterious recessive genes. It was not until the 1960s that the peril of inbreeding in wild species was demonstrated by the considerable fitness cost seen in inbred zoo animals relative to outbred species [1]. Shortly thereafter, ancestral population genetic depletion was uncovered in free ranging species, notable elephant seals in the Pacific, African cheetahs, Florida panthers, Amur tigers, Amur leopards and many others [2–5]. Suddenly, the worry became that intrinsic genetic perils afflicting endangered species would go undetected if population studies were simply gazing through binoculars.

Conservation genetics is now embraced by the genomics community dedicated to widespread sequencing of non-traditional wildlife. Nearly all conservation rescue plans consider genetic and genomic data as an important management component. Broad-based genome sequencing consortia cooperating to achieve whole genome sequence databases of most species have been created. The Earth BioGenome Project serves today as an umbrella organization coordinating the sequence assessment analysis and open release of whole genome sequences for eucaryote taxonomic groups: plants, insects, fungi, marine invertebrates, vertebrates and others [6].

Assessing population genetic diversity reveals a history of population contraction and bottlenecks that lead to genetic reductions in species that were fortunate enough to survive near extinction events. Genome diversity is not the only conservation issue that genomics has informed [2]. New species discoveries plus genetic-affirmed distinctiveness of subspecies also informed wildlife conservation. For example, the definition of explicit tiger subspecies has confirmed the historically separated units of tiger conservation, as well as the postulated founder effect for living tigers caused by the Toba volcanic explosion in Southeast Asia ∼73 000 years ago [4]. Subspecies verification has also been achieved with leopards, pumas, tigers and lions. Population genetic and coalescent dating approaches permit a rough estimation of dates for postulated bottlenecks in the cheetahs, lions, leopards and pumas [3, 5, 7, 8]. Behavioral ecology studies employ genomic methods to affirm adaptive reproductive strategies by kinship and parentage assessment [2]. Emerging pathogens are readily tracked by pathogen sequence analyses defining the dynamics of so many pathogens including HIV-1, SARS and COVID-19 coronavirus outbreaks. Phylogeographic population patterns (defining species and subspecies) plus individual DNA identification have dramatically improved forensic evidence and prosecution in wildlife trafficking cases (e.g Rhino poaching convictions in South Africa).

Since the founding of *GigaScience* a decade ago, multiple conceptual advances have augmented our knowledge of natural history and intervention [9, 10]. Dating methods based on Markov model algorithms are increasingly precise in more refined coalescent timing estimates. Detection of inter-species hybridization illustrates the commonality of gene flow between historic isolated populations and developing species. Such efforts have made the assessment of founder effects, bottlenecks and speciation seem a bit messy, but nonetheless far more precise. Better approaches for estimating the genome-wide genetic load of individuals and populations have inspired population rescue programs to consider the donor populations, increasing our confidence in management of active attempts to rescue and rehabilitate fledgling species [9].

Whole genome sequence from trace amounts of DNA (from scat, hair, saliva, feathers or sloughed skin from sea mammals) has revolutionized the information that genome sequence can reveal, particularly from ancient museum specimens. Signatures of selective adaptation in individual genes are now possible and can be used to consider functional genomic approaches to management. So far, only about 30 species rescue attempts have occurred, some quite successful such as the Florida panther and a few less so [1, 8, 9], For example, the restoration of Isle Royale wolves actually failed because the source population for restoration contained maladaptive alleles camouflaged by chromosome segment heterozygosity [9].

Conservation genomics applications depend more and more on advances in genome bioinformatics programs that automate the cataloging and analyses of genomics data for traditionally unstudied populations. Novel algorithms critically improve estimates of gene flow, migration, coalescent dating, taxonomic distinctions, chromosomal breakpoints, and inferring natural history. But there is a caveat: sequencing and programming mistakes that plague the new bioinformatics tool kits can introduce systematic errors in estimators due to sequence assembly artifacts; hiccups in variant calling; and complex repeat interactions with genome assemblies, alignments, and analyses. A rare mistake in big data covering multi-gigabase genome sequences introduces errors that must be filtered for the dataset to be accurate and applied. Today, a principal concentration of conservation genomics practitioners is quality control of the myriad steps to the genome inferences, conclusions, and interpretation. Finally the sheer complexity of explaining bioinformatics algorithms has led to a reluctance of conservation practitioners—and that must be addressed and ameliorated [10]. Bioinformatics training in Conservation Genomics is a must to persuade conservation practitioners to interpret and unitize the powerful new genomics algorithms and conclusions (see ConGen, the long running course on Recent Advances in Conservation Genetics http://conservationgenetics.org).

## Conclusion

In my travels around the world's pristine places, I always meet dedicated protectors of the wildlife. A common theme heard is that the overwhelming cause of increased species extinction is clearly human associated. We have come a long way in documenting the threats and solutions through multi-disciplinary approaches to threatened fauna and flora. Genomics analysis already helps us more clearly address some of the intrinsic factors that can inform successful conservation intervention. Genome consortia will provide accessible databases for reference genomes of the world's biota within a generation. The computational tools to move big data into standard population analyses are critical for achieving and evaluating derivative results, conclusions and recommended action. Moving from algorithm development to replication, application, confidence, and translation is ongoing at such an exciting time for conservation genomics experts. And it may only be just in time—as the perils and certainty of extinction are marching forward with real immediacy.

## Editors Note

This commentary is part of a series to celebrate a Decade of *GigaScience*, to coincide with the 10th anniversary of our launch in July 2012. These papers take a look back at 10 years of advances in large-scale research as open science has become mainstream. To encourage the use of large-scale genomics data for conservation and increase learning opportunities for women in science from a wide of range of countries, *GigaScience* has been sponsoring young female students from low income countries to attend the international ConGen: Conservation Genetics, Population Genomics, and Molecular Ecology course since 2016.

See more on our sponsoring of ConGen here: http://gigasciencejournal.com/blog/gigascience-supports-congen-2018/.

## Data Availability

Not applicable.

## Competing Interests

The author declares that he has no competing interests.
